# Can Patients Co-Create Value in an Online Healthcare Platform? An Examination of Value Co-Creation

**DOI:** 10.3390/ijerph191912823

**Published:** 2022-10-06

**Authors:** Xiaoyan Ding, Xiang You, Xin Zhang, Yue Yu

**Affiliations:** 1 Library, Shandong Normal University, Jinan 250014, China; 2 China Unicom, Jinan 250014, China; 3 School of Management Science and Engineering, Shandong University of Finance and Economics, Jinan 250014, China; 4 Jinan Urban Development Group, Jinan 250100, China

**Keywords:** value co-creation, information quality, system quality, functional experience, emotional experience

## Abstract

With the development of online healthcare services, patients could receive support and create value with other users on online healthcare platforms. However, little research has been conducted on the internal mechanisms of patient value co-creation from the perspective of online healthcare platforms. To analyze patient value co-creation in online healthcare platforms, this study explores the underlying mechanisms of patient value co-creation among patients. The results show that value co-creation includes patient citizenship behavior and participation behavior. Information quality, peer communication, and system quality influence functional experiences and emotional experiences. In addition, functional experiences and emotional experiences could influence patient value co-creation. This study clarifies the mechanism of value co-creation among patients and provides insight into value co-creation in online healthcare platforms.

## 1. Introduction

With the development of healthcare services, online health platforms provide patients with many functions, such as online consulting and healthcare information support. According to statistics, online healthcare has developed dramatically [1]. Digital products and services will grow to a market share percentage of 12% within the healthcare sector by 2025 [2]. During the COVID-19 crisis, home quarantine was carried out worldwide and patients could not go to hospitals conveniently. Online health platforms have changed the traditional health service modes, optimizing healthcare resource allocation. They provide both patients and doctors with convenient platforms for health information communication, aiding healthcare services and patient visiting processes [3], and promoting information creation and exchange. It could be seen that online healthcare creates value for participators [4], and patients could also obtain and create value in online healthcare platforms. Understanding the internal mechanisms of patient value co-creation is significantly important for online healthcare development and a patient’s health.

In recent years, value co-creation behavior has been investigated comprehensively by industries and scholars [5]. Value co-creation is vital for business revenue and user involvement. In the context of healthcare, with the development of online healthcare platforms, it is assumed that a patient’s value behavior is more complicated [6], which is worthy of deeper investigation. Function, information, and system characteristics could influence how patients behave on online healthcare platforms [7]. In addition, patients could publish personal information, answer questions, and make online reviews, contributing to online healthcare platforms. That is to say, patients can obtain value from online healthcare, and co-create value in online healthcare, thus enriching the online healthcare platform development.

For online healthcare platforms, how to increase the value and maintain the development of the platforms are prominent. Moreover, the patient’s functional and emotional experiences could help to increase positive feelings [8] and further value co-creation. Value co-creation could help to enhance the competitiveness of platforms, promoting further patient value co-creation. It is confirmed that healthcare technology helps co-create value [9]. Social support and technical support are prominent in value co-creation [10]. For patients, online healthcare platforms help obtain healthcare information, save time, and are convenient. Experience in platforms is also important for further usage and value co-creation. In addition, patients could provide healthcare information and publish personal experiences, co-creating value with the platform [11]. That is to say, value co-creation could be achieved by patients and online healthcare platforms. Focusing on patients in online healthcare platforms, the value co-creation by patients and platforms is worthy of further investigation. However, the underlying mechanisms of patient value co-creation lack investigation. Therefore, to explore the mechanisms of patient value co-creation in online healthcare platforms deeply and clearly, this study investigates patient value co-creation from the perspective of an online healthcare platform.

Above all, this study is based on the experiential value theory to investigate the internal mechanisms of value co-creation among patients on online healthcare platforms. From the perspective of platforms, the factors that could influence patient value co-creation are explored comprehensively in online healthcare platforms. It clarifies the internal mechanisms of value co-creation in online healthcare platforms. Therefore, this study helps enrich the investigation of value co-creation among patients on online healthcare platforms, which could also promote the long-term development of online healthcare services.

## 2. Theoretical Background and Literature Review

### 2.1. Experiential Value Theory

Experiential value theory has received much attention in marketing and customer behavior. Notably, value co-creation not only satisfy customers but also benefits enterprises to a large extent. Yi and Gong [12] divided value co-creation into two dimensions—customer participation and customer citizenship behavior. Prahalad and Ramaswamy [13] stressed the role of customer participation in value co-creation. Memory is also worth attention in value co-creation [14]. By providing patients with many services and functions, online healthcare platforms promote communication and are good for value co-creation [10]. Previous study have shown that community support and interaction promote value co-creation [15]. However, the study of value co-creation is mainly investigated in the context of business, virtual communities, and enterprise, but it rarely includes the context of healthcare.

In general, value co-creation could be achieved by patient participation and citizenship behavior. In online healthcare platforms, information seeking and information sharing become prevalent, thus patients could receive help from each other, and platforms could achieve many benefits from participators [6]. It is assumed that much value could be reached through information exchange. Goetzinger [16] found that healthcare information seeking is the main goal of online healthcare platform usage. It is also shown that patients with diseases use online healthcare platforms for disease treatment and prevention [17]. That is to say, value co-creation could be reached by social interaction and information exchange among patients in online healthcare platforms. Thus, the experimental value theory is well suited in the study of value co-creation among patients in online healthcare platforms.

### 2.2. The Characteristics of Online Healthcare Platforms and Patient Experience

Customer experiences are derived from subjective cognition and emotion, and they reflect psychological and behavioral tendencies. Online healthcare has its own special characteristics and it is more complicated than industries and business. Experience is prominent and special in health management. Moreover, regarding some basic functional experiences, such as seeking professional information, patients could also obtain emotional care from the platform. That is to say, in online healthcare, patients could achieve both functional experiences and emotional experiences. It is affirmed that by providing services for users, online healthcare platforms could increase patient experiences to a large extent. For example, some programs have been conducted to enhance patient experiences [8]. In addition, it was shown that patient experiences in online healthcare platforms influence the well-being of patients positively (to some extent) [18].

Patient experiences in online healthcare platforms could be affected by the characteristics of the platform. By providing functions, such as social interactions, online healthcare platforms could influence how patients feel and cognize healthcare. In this study, platform characteristics are divided into information quality, peer communication, and system quality. Recently, the role of patient experience has received much attention [19] and it could be divided into functional experiences and emotional experiences. On the one hand, online healthcare platforms could help healthcare management by providing social functions. In this platform, patients could experience online healthcare services and obtain healthcare information from peer communication. Information quality, system quality, and peer communication reflect platform characteristics, which could increase patient functional experiences and emotional experiences to some extent. On the other hand, from the perspective of patient, patients themselves have different tendencies, and their experiences might vary according to the platform characteristics provided by the online healthcare platform. Therefore, in this study, it is assumed that patient experiences could be influenced by the characteristics of the platform. Thus, it is hypothesized:

**Hypothesis** **1a** **(H1a).** *Information quality could positively influence functional experience*.

**Hypothesis** **1b** **(H1b).** *Information quality could positively influence emotional experience*.

**Hypothesis** **2a** **(H2a).** *Peer communication positively influences functional experience*.

**Hypothesis** **2b** **(H2b).** *Peer communication positively influences emotional experience*.

**Hypothesis** **3a** **(H3a).** *System quality could positively influence functional experience*.

**Hypothesis** **3b** **(H3b).** *System quality could positively influence emotional experience*.

### 2.3. Patient Experience and Value Co-Creation

Patient experiences in online healthcare platforms are of vital importance for disease treatment and prevention [20]. Patient experiences could be divided into functional experiences and emotional experiences. On the one hand, basic functions in online healthcare platforms could satisfy patients’ healthcare needs to some extent. Once patients receive healthcare information, seek professional help, and satisfy their healthcare needs in the online healthcare platform, they have more possibilities to share their information and communicate with others. The functional experiences could promote patients to co-create value in online healthcare platforms. Moreover, to provide feedbacks for others, patients could publish articles, make online reviews, or recommend doctors and hospitals in the online healthcare platform. On the other hand, patients also want to seek emotional support when they seek help online; besides professional knowledge, doctors could give psychological support to patients [21]. It is affirmed that patient ‘reference frames’ on cure and care influence value co-creation [22]. Emotional experiences positively influence perceived value [23].

Notably, patients would be more likely to use online platforms again and contribute more information when their functional and emotional needs are satisfied in online healthcare platforms. That is to say, value co-creation could be increased when patients have better functional and emotional experiences in the online healthcare platform. In this study, patient experiences could be divided into functional experiences and emotional experiences. Value co-creation includes patient citizenship behavior and participation behavior. Therefore, it is assumed that the better patient experience in the online healthcare platform, the more value they could co-create with the platform.

**Hypothesis** **4a** **(H4a).** *Functional experience could positively influence patient citizenship behavior*.

**Hypothesis** **4b** **(H4b).** *Functional experience could positively influence patient participation behavior*.

**Hypothesis** **5a** **(H5a).** *Emotional experience could positively influence patient citizenship behavior*.

**Hypothesis** **5b** **(H5b).** *Emotional experience could positively influence patient participation behavior*.

**Figure 1:**
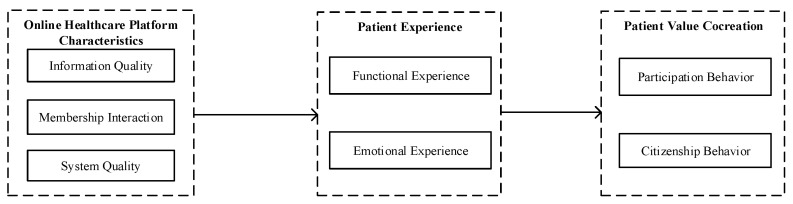
Theoretical Framework.

Above all, the internal mechanism of patient value co-creation needs a comprehensive analysis in the online healthcare platform, therefore, this study builds the theoretical framework shown in Figure 1.

## 3. Materials and Methods

We collected data through online and offline surveys and analyzed them using the structural equation model (SEM). The SPSS (IBM, New York, NY, USA) and Smartpls 3.0 software packages (a private company in Oststeinbek, Germany) were used to evaluate the research model and test hypotheses.

### 3.1. Survey Design

To explore the mechanism of patient value co-creation, we analyzed the research model using survey data. The research model included seven latent variables and the measured items were referenced to previous studies Information quality was measured through four items based on Goetzinger, Park, Jung Lee, and Widdows [16]. Peer communication was measured through four items based on the work of Wang, et al. [24]. System quality was measured through six items from prior studies [25]. The measurements of patient experiences were based on previous studies [26,27], and value co-creation was measured through five items based on previous studies [12,28]. The questionnaire used a seven-point Likert scale, in which one meant strongly disagree and seven meant strongly agree.

### 3.2. Sampling and Data Collection

In this study, we analyzed the mechanism of value co-creation among patients in online healthcare platforms. In other words, the population in our study had experience using online healthcare platforms. Thus, our research context was confined to online healthcare platforms and the respondents were online healthcare platform users. Further, we used some items to demonstrate the constructs we tried to analyze. As original items of constructs were in English, a back-translation method was used to convert these items into Chinese and we made them suitable for the online healthcare context. We discussed the measurement items within our research team (consisting of 3 associated professors and 6 graduate students) to guarantee the content validity, readability, and design quality of the questionnaire. The measurement items were modified according to their suggestions and we reached a final form of the questionnaire. To ensure the reliability and validity of the scales, we selected 31 patients to conduct a pilot test. The results of the test showed that the measurement items were adequate for further implementation. Finally, the questionnaires were distributed in a leading online questionnaire platform in China. This platform is professional for collecting questionnaires and their respondents include people from various backgrounds (including different occupations, ages, education levels, etc.) [29]. Therefore, we distributed questionnaires on this platform and finally received 230 responses. As our target was online healthcare platform users, respondents who were not satisfied were excluded from the final analysis. Thus, our sample was confined to online healthcare users. After eliminating incomplete responses (the same answer for all measurement items and responses with no experience using social media), 205 valid survey responses remained for the final data analysis. Appendix A shows the survey measurement in this study. The demographic characteristics of the sample are shown in Table 1. The online healthcare platforms patients used are shown in Table 2.

## 4. Results

### 4.1. Measurement Model

In this study, we measured the reliability and validity of the constructs. To ensure that the measurements of constructs were suitable, first, we checked the validity of the structural model using KMO and Bartlett’s sphere test. According to previous literature [30], the value of KMO should be above 0.6 and Bartlett’s sphere test should be significant. In a well-designed model, the proportion of cumulative explanatory variables for factors must reach 50%. Our analysis resulted in a KMO of 0.904 and significant Bartlett’s sphere test. The proportion of cumulative explanatory variables for the factors was 62.975%, indicating that the research model was adequate for further analysis.

Secondly, we measured the model’s reliability using Cronbach’s α, composite reliability (CR) and average extraction (AVE), and analyzed the model’s validity through factor loadings, composite reliability (CR), and average extraction (AVE). According to the literature, factor loadings, CR, and Cronbach’s α should be above 0.7, and the value of AVE should be above 0.5. The data analysis indicated that the reliability and validity of the model were adequate (Table 3 and Table 4). Thus, the measurements of constructs in this study were adequate for analysis.

### 4.2. Structural Model

This study also analyzed the path efficient. All our hypotheses are supported. Information quality positively influenced functional experience (β = 0.358, t = 5.502, *p* < 0.001) and emotional experience (β = 0.205, t = 2.626, *p* < 0.05). Thus, H1a and H1b are supported. Peer communication positively influenced functional experience (β = 0.292, t = 4.857, *p* < 0.001) and emotional experience (β = 0.402, t = 6.714, *p* < 0.001); thus, H2a and H2b are supported. System quality positively influenced functional experience (β = 0.138, t = 2.254, *p* < 0.01) and emotional experience (β = 0.219, t= 3.595, *p* < 0.001). Thus, H3b and H3a are supported.

In addition, the patient’s experience could affect the value co-creation behavior. Functional experience could positively influence participation (β = 0.190, t = 2.458, *p* < 0.05) and citizenship behavior (β = 0.313, t = 4.406, *p* < 0.001). Emotional experience positively influenced participation (β = 0.429, t= 6.018, *p* < 0.001) and citizenship behavior (β = 0.183, t = 2.634, *p* < 0.01); thus, H4a, H4b, H5a, and H5b are supported in this study.

Furthermore, to uncover the underlying mechanisms of patient value co-creation more clearly, using mediating analysis, this study analyzed the mediating role of patient experience (functional experience and emotional experience) in the relationship between system characteristics and co-creation behavior. The results show that patient experience partially mediated the relationship between system characteristics (information quality, peer communication, and system quality) and patient value co-creation behavior (participation and citizenship behavior). Thus, patient experience played a mediating role and was vital for the value co-creation in the online healthcare platform. The platform characteristics not only influenced patient value co-creation directly, but also indirectly. The result of mediating analysis could be seen in Table 5, Table 6, Table 7 and Table 8.

## 5. Discussion

Value co-creation has received much attention from scholars and industries. To elicit the generation of patient value co-creation more deeply, this study was based on the experiential value theory, focusing on patient value creation, and exploring the internal mechanism of value co-creation among patients in online healthcare platforms. Previous studies on value co-creation usually focused on e-commerce or social sites; however, there was a lack of attention on online healthcare. Existing studies on value co-creation in healthcare are still in the early stages [31] and have not uncover patient value co-creation from the perspective of online healthcare platforms. Therefore, to analyze the value co-creation among patients more clearly, in this study, the underlying mechanism of patient value co-creation was analyzed comprehensively, in order to help elicit the value co-creation in online healthcare platforms more clearly.

Firstly, it was shown that platform characteristics could influence patient experiences significantly. Consistent with previous studies, online health platforms play vital roles in patient health management [32]. Experiences in online healthcare are influenced by platforms to a large extent [33]. In detail, information quality positively influences a patient’s functional experiences and emotional experiences. It was shown that information could influence a patient’s well-being [34]. Important characteristics of online healthcare platforms involve higher quality information that patients could obtain; more valuable information and emotional care could enhance the patient’s experience. If the platform does not provide users with high quality information, users could decrease their usage of the online healthcare platform, or even discard using the platform permanently. In addition, system quality positively influences functional experience and emotional experience. Stable systems promote patients to have better experiences in online healthcare platforms. Patients could feel better and the functional experiences and emotional experiences could increase. However, uncomfortable systems will not attract users or increase their experiences in the online healthcare platform. Peer communication is a non-neglected factor in patient experiences. Communication plays a prominent role in psychological needs [35]. For patients, besides healthcare needs, well-being plays a vital role in healthcare management. Online healthcare, different from offline channels, provides patients with an online platform for peer communication. Communication with people on an online healthcare platform could make up the healthcare information gap; in addition, patients also satisfy their psychological needs. Thus, peer communication promotes functional experiences and emotional experiences on online healthcare platform.

Secondly, this study found that patient experiences promote participation and citizenship behavior, thus increasing value co-creation on online healthcare platform. The important role of patient experience was also investigated by previous studies [36]. This study is consistent with previous studies in that patients participating in online healthcare platforms are of vital importance for the long-term development of online healthcare platforms [37]. In addition, patients are important members of online healthcare platforms; patient citizenship behavior is beneficial for the online healthcare community construction. With better functions and emotion services provided on online healthcare platforms, patients have more possibilities to show citizenship behavior. Notably, patients are more likely to participate and show citizenship behavior in online healthcare platforms.

Thirdly, using mediating analysis, this study found that patient experiences mediate the relationship between system characteristics (information quality, peer communication, and system quality) and value co-creation behavior (participation and citizenship behavior). Consistent with prior works, the role of experience was also confirmed in this study [8]. Functional experiences and emotional experiences play partial mediating roles in system characteristics and patient value co-creation behavior. From the result, the increase of value co-creation needs better patient experience from the system characteristics perspective. Thus, to promote value co-creation, patient experience is significantly important and worth more attention.

### 5.1. Theoretical Implications

This study has theoretical implications (in three perspectives). Firstly, based on the experimental value theory, this study explores value co-creation among patients and enriches studies on the value co-creation of patients in online healthcare platforms. Value co-creation has huge benefits for the long-term development of healthcare. Current studies on value co-creation are mainly focused on marketing and service industries; however, there is a lack of research on patient value co-creation in online healthcare platforms [19]. This study is based on the experiential value theory; we explored patient value co-creation behavior comprehensively, which could help elicit the underlying mechanism of value co-creation from the context of marketing services to the online healthcare platforms clearly.

Secondly, considering that online healthcare platforms are different from commerce and social networks, this study constructs and broadens the border of value co-creation among patients based on the experiential value theory [38]. Previous studies usually investigated value co-creation from customers and users, but as potential customers in online healthcare platforms, the role of a patient’s value is always neglected. This study broadens the borders of experimental value theory from the perspective of online healthcare platforms, exploring the effects of platform characteristics on patient value co-creation. Therefore, this study uncovers the role of platform characteristics in online healthcare and deepens the value co-creation among patients in online healthcare platforms.

Thirdly, combing functional and emotional characteristics in online healthcare platform experiences, patient experiences were explored in a more comprehensive way in this study [8,20]. This study investigated patient experiences from two aspects—functional experience and emotional experiences, which enrich previous studies on patient experiences from a single perspective in online healthcare platforms [39]. Moreover, the mediating roles of function experiences and emotion experiences are confirmed in the relationship between system characteristics and value co-creation behavior. It could be seen that the functional and emotional experiences of patients are both important in online healthcare platforms, the experiences further make positive effects on patient participation and citizenship behavior. Thus, this study considered the effect of patient experiences in value co-creation, which could help elicit value co-creation more comprehensively.

### 5.2. Practical Implications

Firstly, this study could help online healthcare platforms to co-create value with patients. Patient value co-creation is significantly important for online healthcare development and improvement. By analyzing the internal mechanism of value co-creation in online healthcare platforms, this study could help researchers understand the underlying mechanism of value co-creation and take measures to enhance patient experiences by creating value in online healthcare platforms.

Secondly, this study provides suggestions on characteristics for improvements in online healthcare platforms. Platform characteristics are vital for patient experience and further patient value co-creation. Information quality, peer communication, and system quality are all prominent for platform construction. Online healthcare platforms should try their best to enhance information quality and system quality, promoting peers to communicate fluently in the platform.

Thirdly, patient experiences should be focused on when providing online healthcare services. Functional experiences and emotional experiences reflect patient experiences in online healthcare platforms. In addition, patient experiences could influence value co-creation positively. Functional experiences and emotional experiences promote patients to participate in online healthcare, and patients could show citizenship behavior when they have excellent experiences in online healthcare platforms. Thus, enhancing patients’ functional and emotional experiences are of vital importance for online healthcare platform development.

## 6. Conclusions

This study explores the internal mechanism of value co-creation among patients in online healthcare platforms. It was shown that information quality, peer communication, and system quality could influence functional experiences and emotional experiences, which could further promote value co-creation among patients in online healthcare. This study enriches the investigations on value co-creation in online healthcare platforms, promoting online healthcare services. However, there are some limitations. Firstly, this study was limited to online healthcare platforms, and there needs to be a deeper analysis on other contexts to explore patient value co-creation more comprehensively. Neutrosophic statistics could help improve the study performance and lessen uncertainty [40]. It is assumed that neutrosophic statistics could be used for future research to decrease uncertainty in this study [41]. Secondly, this study does not consider the differences of patient value co-creation between males and females, which could not broaden the application to certain populations. In addition, other factors, such as national policies, were not included when investigating the mechanism of patient value co-creation; thus, factors from other perspectives should also be considered in future research studies. Thirdly, the role of platform design could not be neglected; thus, co-designs with patients in online healthcare platforms should be promoted and future studies could explore value co-creation from multiple perspectives.

## Figures and Tables

**Table 1:** Demographic.

Variable	Category	Number	Percentage
Gender	Male	96	46.8%
	Female	109	53.2%
Age	Less than 25	49	23.9%
	26–30	75	36.6%
	31–40	58	28.3%
	41–50	19	9.3%
	Above 50	4	2.0%
Education	Senior high school and lower	3	1.5%
	Specialty	36	17.6%
	Bachelor	96	46.8%
	Master and higher	70	34.1%

**Table 2:** Summary of online healthcare platform.

Name	Number	Percentage
Haodf	120	58.5%
Chunyu Doctor	51	24.9%
Pinan Doctor	74	36.1%
Pinan Doctor	76	37.1%

**Table 3:** Reliability and validity.

Variable	Item	Factor Loading	Cronbach’s α	CR	AVE
Information Quality	HI1	0.915	0.957	0.967	0.852
	HI2	0.930			
	HI3	0.932			
	HI4	0.924			
	HI5	0.916			
Peer Communication	CQ1	0.921	0.957	0.969	0.886
	CQ2	0.955			
	CQ3	0.945			
	CQ4	0.943			
System Quality	SQ1	0.960	0.945	0.965	0.901
	SQ2	0.945			
	SQ3	0.943			
Functional Experience	FE1	0.939	0.958	0.969	0.887
	FE2	0.948			
	FE3	0.949			
	FE4	0.930			
Emotional Experience	EE1	0.957	0.967	0.976	0.909
	EE2	0.953			
	EE3	0.952			
	EE4	0.952			
Participation	PB1	0.956	0.977	0.982	0.915
	PB2	0.951			
	PB3	0.962			
	PB4	0.961			
	PB5	0.953			
Citizenship Behavior	CB1	0.938	0.964	0.972	0.874
	CB2	0.940			
	CB3	0.923			
	CB4	0.954			
	CB5	0.920			

**Table 4:** Discriminant validity.

Variable	HI	CQ	SQ	FE	EE	PB	CB
HI	0.923						
CQ	0.176	0.941					
SQ	0.280	0.294	0.949				
FE	0.337	0.396	0.324	0.942			
EE	0.448	0.502	0.394	0.445	0.953		
PB	0.364	0.381	0.382	0.382	0.514	0.957	
CB	0.294	0.340	0.280	0.395	0.323	0.301	0.935

**Table 5:** The mediating roles of functional experiences in the relationships between system characteristics and participation.

IV	Mediation Path	Effect	Coefficient		Bias-Corrected		Percentile	
			SE	t	95% CI		95% CI	
HI	Direct	0.242	0.071	3.422	0.103	0.381	0.103	0.381
CQ	Indirect	0.122	0.044	2.773	0.053	0.229	0.047	0.222
SQ	Direct	0.273	0.068	4.002	0.138	0.407	0.138	0.407
FE	Indirect	0.108	0.038	2.842	0.047	0.207	0.041	0.195
EE	Direct	0.289	0.066	4.392	0.159	0.418	0.159	0.418
PB	Indirect	0.093	0.034	2.735	0.041	0.180	0.035	0.168

**Table 6:** The mediating roles of emotional experiences in the relationships between system characteristics and participation.

IV	Mediation Path	Effect	Coefficient		Bias-Corrected		Percentile	
			SE	t	95% CI		95% CI	
HI	Direct	0.215	0.062	3.449	0.092	0.338	0.092	0.338
CQ	Indirect	0.149	0.044	3.386	0.074	0.256	0.065	0.245
SQ	Direct	0.164	0.069	2.387	0.029	0.300	0.029	0.300
FE	Indirect	0.216	0.059	3.661	0.118	0.354	0.113	0.344
EE	Direct	0.212	0.064	3.311	0.086	0.338	0.086	0.338
PB	Indirect	0.170	0.043	3.953	0.098	0.269	0.088	0.261

**Table 7:** The mediating roles of functional experiences in the relationships between system characteristics and citizenship behavior.

IV	Mediation Path	Effect	Coefficient		Bias-Corrected		Percentile	
			SE	t	95% CI		95% CI	
HI	Direct	0.147	0.072	2.058	0.062	0.288	0.062	0.288
CQ	Indirect	0.147	0.041	3.386	0.073	0.234	0.070	0.232
SQ	Direct	0.216	0.069	3.142	0.081	0.351	0.081	0.351
FE	Indirect	0.122	0.034	3.588	0.065	0.207	0.062	0.197
EE	Direct	0.169	0.067	2.514	0.037	0.302	0.037	0.302
PB	Indirect	0.110	0.028	3.929	0.061	0.177	0.057	0.173

**Table 8:** The mediating roles of emotional experiences in the relationships between system characteristics and citizenship behavior.

IV	Mediation Path	Effect	Coefficient		Bias-Corrected		Percentile	
			SE	t	95% CI		95% CI	
HI	Direct	0.209	0.069	3.022	0.073	0.346	0.073	0.346
CQ	Indirect	0.085	0.030	2.833	0.035	0.164	0.028	0.147
SQ	Direct	0.236	0.075	3.133	0.087	0.384	0.087	0.384
FE	Indirect	0.102	0.042	2.429	0.035	0.206	0.027	0.201
EE	Direct	0.180	0.071	2.521	0.039	0.321	0.039	0.321
PB	Indirect	0.099	0.033	3.000	0.043	0.178	0.039	0.170

## Data Availability

Not applicable.

## Appendix A

1. Your gender:
  ○ Male  ○ Female
2. Your age:
  ○ 18 years old or lower  ○ 18–25 years old  ○ 26–30 years old
  ○ 31–40 years old  ○ 41–50 years old
  ○ 51–60 years old  ○ 60 years old
3. Your highest education:
  ○ High school/technical secondary school and below
  ○ Junior college
  ○ Bachelor’s degree
  ○ Master’s degree or above
4. Your current occupation:
  ○ Students in school
  ○ Civil servant
  ○ Ordinary workers
  ○ Researchers
  ○ Professionals and technicians
  ○ Freelancers
  ○ A farmer
  ○ Other
5. Your current monthly income:
  ○ 3000 and below
  ○ 3000–5000
  ○ 5000–8000
  ○ 8000–10,000
  ○ 10,000 and above
6. The Internet healthcare platform you have used:
  □ Haodf
  □ Chunyu doctor
  □ Dingxiang doctor
  □ Pingan doctor
  □ Medical Association Shandong
  □ ________
7. The purpose of using online healthcare platform [Multiple choice]:
  □ Understand medical and health information
  □ Online consultation
  □ Telephone Consultation
  □ Online reservation
8. How often do you use the online healthcare platform:
  ○ Once or more times a week
  ○ Once a month
  ○ Once every two months or more

The following questions should be rated in 7-Likert scale [30].

9. In the online healthcare platform, there is a large number of patient evaluations of doctors’ medical levels and service attitudes.
10. In the online medical service platform, there is a large number of patient experiences on disease prevention, diagnosis, and treatment.
11. In the online healthcare service platform, there is a large number of identical and complementary healthcare information.
12. I often communicate and interact with doctors in the online healthcare platform.
13. I often communicate and share information with other patients in the online healthcare platform.
14. I would use the functions in the online healthcare platform (online reservation, information search, online consultation, etc.) frequently.
15. In the online healthcare platform, I often browse the content provided by various service sections.
16. The operation is simple and fast in the online healthcare platform.
17. It is easy for me to do what I want in the online healthcare platform.
18. The interface of the online healthcare platform is beautiful and simple.
19. In the online healthcare platform, the medical information provided is reliable and trustworthy.
20. The online healthcare platform provides me with authoritative service information and professional information of doctors.
21. The online healthcare platform provides me with the latest medical information.
22. In the online healthcare platform, the prices of online doctor-patient communication and telephone consultation services are acceptable.
23. The quality of online healthcare information affects my healthcare perception.
24. In the online healthcare platform, online medical information and online consulting services can reduce my blindness.
25. In the online healthcare platform, my privacy is protected, and the functions and services will not reveal my personal information.
26. The online healthcare platform reduces my time cost of seeing a doctor.
27. Online healthcare platform reduces my efforts to find quality medical services.
28. The online healthcare platform enables me to obtain more cost-effective medical services.
29. When I use this online healthcare platform, I pay attention to the information posted by users (doctors, patients).
30. When I use the online healthcare platform, I am willing to share my medical information with others.
31. After using the online healthcare platform, I have formed my own medical knowledge and will accept, recognize and understand my actual condition.
32 I have a close relationship with the users (doctors, other patients) on the online healthcare platform.
  I interact a lot with users on the platform (doctors, other patients).
33. I will recommend the online healthcare platform to my friends and relatives.
34. After using the online healthcare platform, I will make positive comments on the service.
35. I am willing to put forward suggestions to the online healthcare platform to improve the service.
36. Due to special reasons for service problems, I will choose to forgive the online healthcare platform.
